# Porous Silicon Optical Biosensors: Still a Promise or a Failure?

**DOI:** 10.3390/s19214776

**Published:** 2019-11-03

**Authors:** Luca De Stefano

**Affiliations:** Institute for Microelectronics and Microsystems, National Research Council, Via P. Castellino 111, 80131 Napoli, Italy; luca.destefano@cnr.it

**Keywords:** porous silicon, optical biosensors, functionalization, label-free imaging

## Abstract

Even if the first published article on a porous silicon (PSi)-based biosensor dates back to more than twenty years ago, this technology still attracts great attention from many research groups around the world. In this brief review, the pros and cons of porous silicon-based optical biosensors will be highlighted on the basis of some recent results and published papers on this subject. The aim of the paper is to give a straightforward introduction to PhD students and young researchers on this subject, which is particularly full of educative content, since it is highly multidisciplinary. Fabrication of PSi-based optical biosensors requires competencies related to many different scientific topics ranging from material science, physics and optics to healthcare and environmental monitoring through surface chemistry and more.

## 1. Introduction

When I was offered the chance to give a lecture on optical biosensors based on porous silicon (PSi) at the prestigious ASCOS 2019 school, dedicated to optical biosensing for life science and environmental applications, I immediately asked myself if the topic, as well as being one I was really confident in, was innovative enough to be presented properly to an audience of students from all over the world. This doubt arose from the observation that the first article on PSi-based optical biosensors was published more than twenty years ago [1], and it was therefore legitimate to ask myself the question as to whether this argument was still relevant. The answer was that it was certainly worth, it given the large interest that this technology still arouses in many laboratories scattered all over the world, from Europe to Australia, from China to America. In the last five years, more than 400 scientific papers have been published dedicated to optical sensors in porous silicon in peer-review journals (number estimated by Scopus, accessed in July 2019); a number that is not very high in absolute terms, but which testifies to the liveliness of the scientific community in relation to this topic.

PSi is fabricated by the electrochemical dissolution of doped crystalline silicon using a hydrofluoric-based water solution. The dissolution of the silicon is mediated by the charge carriers, i.e., the electrons and holes, and proceeds from the surface in contact with the solution down by a self-stopping mechanism, so that complex multi-layered structures can be realized by a single computer-controlled etching procedure without touching the sample. All these steps are clearly described in the recent book by Prof. M.J. Sailor at the University of California San Diego, USA, which leads one of the most active groups in PSi-based research topics [2]. On the webpage of Sailor’s group, there are also illustrative videos on PSi fabrication and characterization [3]. The realization of optical PSi structures could be considered simple, but it is not trivial, and a lot of attention should be paid in order to obtain regular and repeatable devices with high performance capabilities. The porosity is one of the main characteristics of PSi films. Porosity is defined as the amount of void in a solid layer, and it is related to the number and the size of the pores created by the dissolution of the bulk silicon. In principle, its value could range between 0 (no dissolution of crystalline silicon) and 1 (all air). By tuning the porosity during the electrochemical etching, by changing the time length and the current flowing into the cell, the refractive index of a PSi layer can be changed from that of the silicon (more than approximately 3.88 in the visible wavelength interval) to that of air (1, by definition). Simple homogeneous PSi layers, as well as very complex multilayered ones, such as Fibonacci and Thue-Morse sequences, can be realized just by writing different “recipes” for the computer-aided voltage controller [4,5]. Similar optical structures could of course be realized by classic micro/nanofabrication techniques, such as those for thin film deposition, i.e., chemical vapor deposition and correlated processes. However, the optical feature is not the only one required in a transducer material for sensing applications. There are many other issues that must be correctly addressed. In the following, the main pros and cons of PSi as a nanostructured material for optical biosensors will be considered and critically analyzed.

## 2. The Pros of PSi-Based Optical Biosensors

An optical transducer is, by definition, a device made of a material that is light active. This means that, after the molecular interaction between the probe and the target, some feature of the light coming from or through the transducer changes as much as possible. The absorption, the phase, the intensity, or whatever other property of the propagating light is therefore monitored and correlated with the biomolecular event under investigation. The greater the variation, the better the transducer. Things work even better if there is a resonance in the optical spectrum; for example, a peak in the absorbance or a dip in the transmittance. A narrow resonance is simpler to follow during the evolution of the optical signal, and thus the sensitivity of the method could increase. All optical and photonic devices for biosensing are designed so that the sensitivity is maximized, and the limit of detection can be as low as possible [6]. The realization of resonant photonic structures based on crystalline silicon or III-V semiconductors requires accurate numerical calculation and the best of the available technologies for micro- and nano-fabrication. Moreover, all of this instrumentation must be placed in a special environment, i.e., a clean room, where temperature, humidity and dust concentration are carefully controlled. All of these restrictions have made integrated photonic devices for biosensing available to only few teams around the world, and at costs that are unsustainable for small research groups. PSi photonic structures can be fabricated under a chemical hood even outside a clean room, with a simple electrochemical cell and a current-voltage controller. The investment required for this equipment is within the reach of almost all laboratories that deal with material science. Furthermore, the technical skills for PSi fabrication can be learned by anyone with a minimal background in chemistry [7], even if the handling of HF, which is a very dangerous substance, requires a proper specific course of instruction regarding safe practice with this reagent. High-sensitivity PSi optical sensors have recently been published by the group of Prof. G. Barillaro at the University of Pisa, Italy [8,9]. The PSi-based photonic crystal realized demonstrated a sensitivity of 1000 nm/refractive index unit, which was able to compete with the better-integrated optical devices, such as nanorings or nanopillars. Its low cost and simple (but not trivial) fabrication procedures are certainly pros in favor of PSi. 

**Figure 1:**
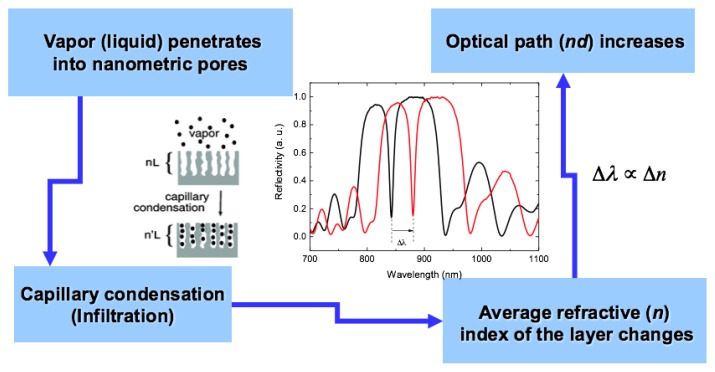
Schematic representation of PSi sensing mechanism based on the variation of the average refractive index.

How does a PSi optical transducer work? Figure 1 shows a simple scheme for this well-known mechanism.

Every substance that penetrates into the sponge-like matrix of a PSi layer changes its average refractive index so that the optical spectrum also changes. The shift of the optical spectrum depends on two parameters, one being the pore-filling ability, the other being the value of the refractive index. The first is strictly determined by the chemical nature of the substance, such as hydrophilicity or hydrophobicity, viscosity and so on; while the second is an intrinsic characteristic of the substance considered. This is the reason a PSi device is only a specific optical sensor, i.e., it gives different responses to different substances, but is not selective, which means that it would not be able to recognize a component in a complex mixture [10,11].

**Figure 2:**
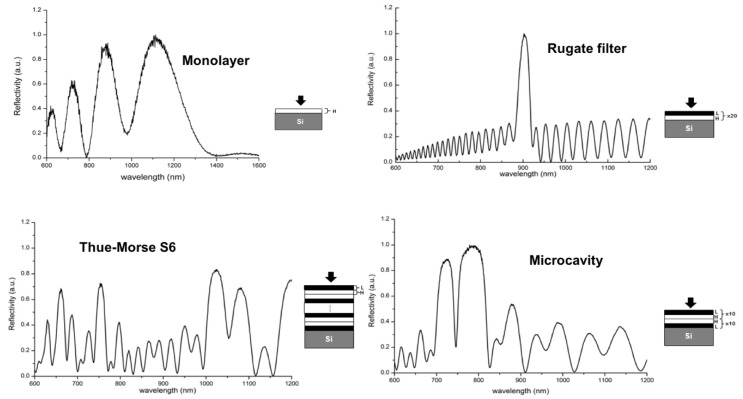
Optical spectra of different PSi optical structures. H stands for the high porosity layer, L for the low porosity layer. The numbers indicate how many pairs of H-L layers constitute each structure.

Anyone confident with electrochemical processes, such as electrodeposition or electro-dissolution of bulky materials, knows that surface roughness can be a serious issue. The optical signal quickly degrades during the propagation inside or through such materials, and this effect can prevent their use as optical transducers. Another impressive feature of PSi optical chips is their very good signal quality. When fabricated on chip, the PSi photonic structure read out in reflection is very simple. As shown in Figure 2, a white light was shone onto the chip and the reflected beam is sent to an optical spectrum analyzer by an optical fiber. There is no need for either critical alignment procedures or sophisticated optical components; another important pro in favor of porous silicon. From homogeneous layers, which optically act as a Fabry-Perot interferometer, to complex sequences of layers having different thicknesses and refractive indexes, such as Bragg mirrors or optical microcavities or Thue-Morse sequences, the optical spectrum is not noisy and its evolution during the experiment can be used to quantify chemical and biological interactions that happen on PSi surface. More simply, especially in time-resolved measurements, the PSi components can be read out at a single wavelength by using a monochromatic source (a laser or a LED) and a photodetector. Remaining precisely on this point, the time response of an optical PSi sensor is very fast; on the order of microseconds [12,13]. As a matter of fact, changes in the refractive index can be revealed immediately, whereas the electrical counterparts are linked to the transit time of the charge carriers [14]. The response time can be further reduced when the optically active PSi elements are integrated into microfluidic systems that increase their time performances by reducing the volume of the sample used [15]. Integrability and fast response can also be ascribed to the pros of PSi.

In addition to the transducer material, the other key issue in a biosensor is the molecular probe, i.e., the natural (such as antibodies, DNA fragments, proteins, enzymes, and so on) or synthetic (like aptamers, PNA, short chains, and others) element that has the role of selectively recognizing the target analyte. All of these probes evolved or have been designed to work in solution, rather than when fixed on a support surface. The functionalization of the transducer surface is a crucial point in realizing an effective device. There are really a lot of chemical and physical strategies for modifying the surfaces of optical devices. The two main options are the spontaneous absorption of biomolecular probes onto the transducer surface, which gives a disordered assembly and an incompletely covered support; or the covalent binding of the probes to surface, which is a much more controlled process. In both the cases, a thin layer of a few nanometers of biological material that is not densely packed should be carefully analyzed in order to design the biosensor performances. On a planar surface, this is not simple, and sophisticated techniques such as X-ray photoelectron spectroscopy must be used. The situation is much easier in the case of PSi structures, which always have very large specific surface area that amplifies the effect of the functionalization treatment. From this point of view, a PSi layer is the perfect support for the development and optimization of a surface functionalization process [16]. The as-etched PSi surface is highly hydrophobic, due to the acid-based fabrication, but it can easily be turned into a hydrophilic one through several oxidation treatments. Moreover, there is at least one procedure that transforms PSi into an ultra-stable material: thermal acetylation, which was discovered by J. Salonen at University of Turku, Finland [17]. The tunable surface chemistry makes PSi devices more useful than those made in planar configuration.

In summary, a high-quality, fast, and specific optical response, a great surface area (up to hundreds of square meter per gram) due to the porous morphology, and a customizable surface chemistry a great flexibility to PSi biosensors for very different applications in environment or healthcare monitoring.

## 3. The Cons of PSi Technology

After listing all of the advantages of this material, it is natural to wonder why it is still not possible to find devices based on porous silicon in the pharmacy or among consumer electronics products. Let’s now face the main issues that until now have prevented the diffusion of PSi-based products onto the market. The doping level and the hydrogen contamination of crystalline silicon wafers can be different from one pack to another, and even in the same lot of wafers, so that the same fabrication recipe applied to two different silicon wafers can generate quite different PSi structures. In particular, hydrogen contamination is responsible for the presence of a top layer, near the wafer surface, with very small pores (under 5 nm) that prevent the penetration of liquids inside the PSi. This top layer can be avoided by removing a few microns of silicon by controlled electropolishing before starting the electrochemical etching of the designed structure. Furthermore, it should be noted that even if the geometry of the platinum cathode should guarantee a homogeneous dissolution rate of the entire silicon surface, this does not actually happen. There is always a small difference in the porosity and thickness values between the edge of the PSi area and its center, and also between the top and the bottom of the layer [18]. This inhomogeneity is reflected in the optical spectrum, which can be different when recorded at different points of the device’s surface. From this perspective, a PSi optical transducer could show different signals from point to point, and, moreover, different spectra from sample to sample. The large-scale industrial production of PSi-based devices is seriously hampered by the intrinsic limitation of the fabrication method. Even if all the steps, from the electrochemical etching to the bioprobe binding, could be individually calibrated, the sum of all of them remains unpredictable, so that the only way to use a PSi biosensor is to calibrate each single device. This solution could be economically sustainable only for those applications with a very high added value [19,20].

The freshly etched PSi surface is highly hydrogenated, and since the chemical bond between silicon and hydrogen (namely, Si-H) is not thermodynamically very stable, it tends to be substituted by Si-O on exposure to atmospheric oxygen. The silicon oxide has a refractive index value much lower than crystalline silicon, such that the spontaneous aging of the porous silicon surface strongly affects the optical spectrum, and also compromises the complex multilayered structures. There are a lot of procedures for passivating and stabilizing the PSi surface, but the complete chemical stability in either acid or oxidizing solution is hard to obtain [21]. This is another difficult issue to overcome that sometimes precludes good repeatability among the results of different experiments obtained using the same device.

## 4. Conclusions and Further Perspectives

Three review papers regarding PSi-based optical biosensors have recently been published, and they give a wide and complete overview of this subject, along with the references reported therein [22,23,24]. In particular, the paper by N.H. Maniya reports a very interesting summary of the detection of various biomolecules using PSi optical devices. An extract of that summary is reported in Table 1 for the purposes of discussion.

The performances of PSi-based optical biosensors reported in Table 2 are equivalent to or exceed those of corresponding devices made of other materials, as reported in the most current literature [29,30,31].

Despite these results, there is no doubt that the most interesting prospect in the field of technological applications of PSi, and also that with the greatest marketing possibilities, is not that of devices made on chips, but rather that of silicon nanostructured powders. Nevertheless, it can be noted that the advantages of using PSi as a material for the optical transduction of molecular interactions largely overcome the disadvantages, as is clearly shown in Table 2.

So many good features in a single material greatly justify the success and the diffusion of PSi in the laboratories focused on optical biosensing that are spread all over the world. The PSi scientific community is very active and collaborative and often meets at international congresses dedicated to the science and technology of porous semiconductors. 

In conclusion, there are strong indications in the direction of constant attention being directed towards the development of PSi biosensors by several academic groups in the mid-term, i.e., in the next five years. The final approach of these products to the commercial market will also depend on a variety of factors directly connected to economic and financial aspects, which can in future change the trends and determine the success of industrial initiatives. PSi optical transducers are thus surely not a failure from a scientific point of view, and they could become successful devices in the near future, even if more effort needs to be directed towards their development.

## Figures and Tables

**Table 1:** Examples of detection of different biomolecules using PSi optical biosensors. Adapted from Ref. [24].

Analyte	Probe	PSi Device	Detection Range	Sensitivity	Response Time	Ref.
DNA (15 mer)	ssDNA	Single layer	1–10 nM	1 nM	20 min	[25]
Metallo-proteinase	Peptide	Microcavity	10^−7^–10^−12^ M	10^−19^ M	15 min	[26]
Subtilisin	Gelatin	Bloch surface wave	0.01 mg/mL	1.8 pM	20 min	[27]
Vancomycin	Peptide	Double layer	0.005–0.1 mg/mL	0.005 mg/mL	20 min	[28]

**Table 2:** Summary of pros and cons of PSi technology.

Pros	Cons
Low costs and simple (but not trivial) fabrication equipment and procedures	Intrinsic limitation of the etching process
Easy and fast read out	Spontaneous aging and chemical instability
Integrated microelectronics compatibility	Single device calibration
Tunable morphology, dielectric properties and surface chemistry	
Huge specific surface area	
Biocompatibility	
Extreme flexibility in different application fields	
